# Likely Pathogenic Variants in One Third of Non-Syndromic Discontinuous Cleft Lip and Palate Patients

**DOI:** 10.3390/genes10100833

**Published:** 2019-10-22

**Authors:** Bénédicte Demeer, Nicole Revencu, Raphael Helaers, Cica Gbaguidi, Stéphanie Dakpe, Geneviève François, Bernard Devauchelle, Bénédicte Bayet, Miikka Vikkula

**Affiliations:** 1 Human Molecular Genetics, de Duve Institute, University of Louvain, 1200 Brussels, Belgium; demeer.benedicte@chu-amiens.fr (B.D.); nicole.revencu@uclouvain.be (N.R.); raphael.helaers@uclouvain.be (R.H.); 2 Center for Human Genetics, CLAD Nord de France, CHU Amiens-Picardie, 80054 Amiens, France; 3 Université Picardie Jules Verne, EA CHIMERE, EA 7516, 80054 Amiens, France; dakpe.stephanie@chu-amiens.fr (S.D.); devauchelle.bernard@chu-amiens.fr (B.D.); 4 Facing Faces Institute, 80054 Amiens, France; 5 Center for Human Genetics, Cliniques universitaires Saint-Luc, University of Louvain, 1200 Brussels, Belgium; 6 Department of Maxillofacial Surgery and Stomatology, Centre de Compétence Fentes et Malformations Faciales (MAFACE), CHU Amiens-Picardie, 80054 Amiens, France; 7 Department of Pediatrics, Cliniques Universitaires Saint-Luc, University of Louvain, 1200 Brussels, Belgium; genevieve.francois@uclouvain.be; 8 Centre Labiopalatin, Division of Plastic Surgery, Cliniques Universitaires Saint-Luc, University of Louvain, 1200 Brussels, Belgium

**Keywords:** discontinuous cleft, *FGFR1*, *DLG1*, WES (whole exome sequencing)

## Abstract

Oral clefts are composed of cleft of the lip, cleft of the lip and palate, or cleft of the palate, and they are associated with a wide range of expression and severity. When cleft of the palate is associated with cleft of the lip with preservation of the primary palate, it defines an atypical phenotype called discontinuous cleft. Although this phenotype may represent 5% of clefts of the lip and/or palate (CLP), it is rarely specifically referred to and its pathophysiology is unknown. We conducted whole exome sequencing (WES) and apply a candidate gene approach to non-syndromic discontinuous CLP individuals in order to identify genes and deleterious variants that could underlie this phenotype. We discovered loss-of-function variants in two out of the seven individuals, implicating *FGFR1* and *DLG1* genes, which represents almost one third of this cohort. Whole exome sequencing of clinically well-defined subgroups of CLP, such as discontinuous cleft, is a relevant approach to study CLP etiopathogenesis. It could facilitate more accurate clinical, epidemiological and fundamental research, ultimately resulting in better diagnosis and care of CLP patients. Non-syndromic discontinuous cleft lip and palate seems to have a strong genetic basis.

## 1. Introduction

Cleft of the lip and/or palate (CLP) are among the most common birth defects, with an approximate incidence of 1/700 live births and with a wide variability of expression depending on ethnicity, gender and cleft type. Although CLPs are not associated with an elevated rate of mortality in developed countries, they represent a significant lifelong morbidity. This requires multidisciplinary treatment and care.

CLPs are classified as syndromic and non-syndromic (isolated). The latter is not associated with additional developmental, structural and/or cognitive anomalies. Syndromic CLPs, which represent 30% of all CLPs, generally follow a Mendelian inheritance with an important variability in expressivity and incomplete penetrance. The other 70% of CLPs occur as isolated lesions and are considered to have a complex etiology with both genetic and environmental factors acting in concert. 

Given the complex and heterogeneous nature of non-syndromic clefts, different genetic approaches have been employed successfully to identify chromosomal loci and genes [1]. With rapid improvement in technology, in particular the development of next-generation sequencing and bioinformatic algorithms, we now have the ability to interrogate the entire exome or genome in order to identify risk loci [2]. Whole exome sequencing (WES) has been successfully performed for Mendelian disorders, and is becoming a useful approach for proving clinical molecular diagnosis [3]. This strategy has also been applied to identify the causes of complex traits including cleft lip and palate [4,5].

Historically, CLP has been divided into cleft of the lip (CL), CL with cleft of the palate, which are often co-classified as cleft of the lip with or without cleft palate (CL/P), and cleft of the palate (CP). This historical broad subdivision is based on a number of epidemiological features of each condition, and a distinct developmental origin of the primary palate and the secondary palate. Segregation of CL/P and CP has been exceptionally reported for families with etiologic mutations in specific genes: *P63, MSX1, IRF6,* and *FGFR1* [1]. Clefts of the lip and palate show a range of phenotypic expression, and dividing CLP in a simplistic way to CL/P and CP has the potential to lose important information. Separately analyzing CLs from the rest of the CL/P group in non-syndromic multiplex families revealed differences in linkage results [6]. It highlights the importance of sub-phenotyping, and the requirement of carefully stratified cohorts to further understand the genetic heterogeneity underlying non-syndromic CLP (nsCLP).

**Figure 1:**
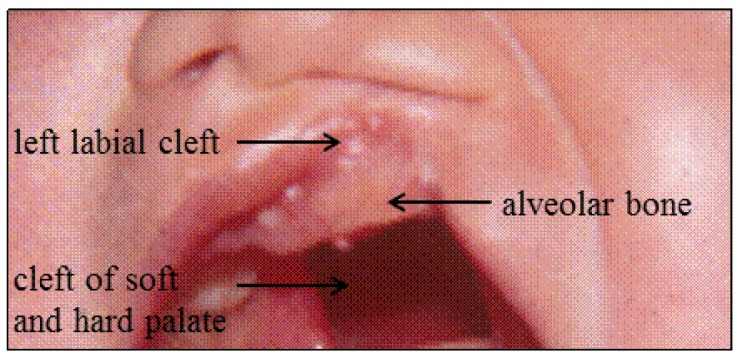
Example of a discontinuous cleft of the lip and/or palate (CLP) with left labial cleft associated with a cleft of the soft and hard palate. Note the presence of alveolar bone.

One overlooked sub-phenotype within CL/Ps is the so called “discontinuous cleft”, which associates a discontinuity in clefting of the primary palate and of the secondary palate (Figure 1). Concerning the description of this CLP, some authors use the term “primary palate” to refer to the anterior intermaxillary palate, whereas others use it to describe the tissues formed by the fusion between the maxillary and medial nasal processes: the lip, alveolus and anterior intermaxillary palate [7]. We use the term discontinuous cleft for any cleft of the primary palate respecting at least part of the intermaxillary palate associated with any cleft of the secondary palate. This atypical phenotype has been reported in the literature with diverse terminology, referred to as “separate cleft of the lip and palate”, “unconnected cleft”, “bridged cleft” and “interrupted cleft” [8,9]. It has been seldomly separated from other subtypes, and likely, if not specified, included either in the CL or CL/P group. A recent report on 356 Brazilian individuals with discontinuous cleft estimated that this group represents 5% of CLP [8]. The pathophysiology behind this subgroup is unknown. In this study, we conducted WES and apply a candidate gene approach on seven individuals with non-syndromic discontinuous CLP in order to identify genes and deleterious variants that contribute to this phenotype.

## 2. Materials and Methods

### 2.1. Patient Selection

Clinical data and samples from patients with nsCLP and their family members were collected in collaboration with the Centre Labiopalatin, Cliniques Universitaires St Luc, Brussels, Belgium and Amiens-Picardie Hospital, France. Informed consent was signed by each participant prior to conducting the study, as approved by the institutional review board. For each patient, a standardized questionnaire was filled in, notably detailing family history and clinical phenotype. The CLP phenotyping was based on clinical examination by the plastic surgeon while evaluating the infant before surgery and follow up, and on the questionnaire. Eight patients who met the clinical diagnosis of non-syndromic discontinuous CLP were selected and included in the study. This comprises 6 sporadic cases and 1 with a familial history of CL/P (Table 1).

### 2.2. Whole Exome Sequencing

DNA was extracted from blood samples using the Wizard® Genomic DNA Purification Kit (Promega). WES was carried out using 1 μg of genomic DNA per sample. Exomes were captured using a commercial enrichment kit (Agilent SureSelectXT Human All Exon KitV5) and enriched libraries were sequenced on an Illumina Hiseq2000. Generated reads were aligned to the human genome build hg19 using Burrows-Wheeler Aligner (BWA) 0.7.15. Data processing and variant calling were performed with Picard 1.107, Genome Analysis Toolkit 3.3 package (GATK), respectively. The generated variant files (.vcf) were imported into a database and further analyzed using Highlander 14.10.3, an in-house bioinformatic framework for variant annotation, filtering, and visualization [10].

We considered rare variants as those with a frequency below 0.5% in public databases: Genome Aggregation Database (gnomAD) [11], Exome Aggregation Consortium database (ExAC) [12], 1000 Genomes [13] and Genome of the Netherlands (GO-NL [14]) and in our in-house control database. As “likely pathogenic“ variants, we considered those with a high impact (canonical splice-site variants and those generating a premature termination codon = PTC (Premature Termination Codon: out-of-frame indels and nonsense variants), or a moderate impact (non-synonymous), as annotated by SnpEff 4.1 and a combined annotation dependent depletion (CADD) PHRED score >15. Missense variants were considered as possibly pathogenic only if predicted to be possibly/probably damaging in at least three out of seven in silico variant classifiers (Sift, Polyphen, Likelihood Ratio Test (LRT, Mutation Taster, Mutation Assessor, Functional Analysis through Hidden Markov Models (FATHMM, DEOGEN (a variant effect predictor that could handle both missense SNVs and in-frame INDELs)). To maximise the conclusion that a damaging variant will be causal, we focused on a list of 89 genes implicated in both syndromic and non-syndromic CLP [15], nsCLP candidate genes [16] and a selection of CLP candidate genes that we gathered from publications [17,18,19,20,21,22,23,24,25] (Supplementary Table S1).

For all variants that satisfied our criteria, we performed co-segregation analysis on all the affected and non-affected family members that were available. Variants were PCR-amplified followed by sequencing on an ABI 3130XL genetic analyzer (Applied BioSystems, city, country).

## 3. Results

Using our filters and candidate gene list, we were able to identify three genetic variants that we consider as causative of the respective phenotype. They are all consistent with a dominant inheritance with an incomplete penetrance model. They generate premature stop codons in genes that have already been linked to a human CLP phenotype and/or syndrome.

### 3.1. Patient F1-3

The proband is the only child of an unrelated Caucasian couple with an unremarkable family history. She was born at 37 weeks of gestation (WG) with birth weight BW = 3070 g and birth height BH = 47 cm. She had a right labioalveolar cleft lip associated with a submucous cleft. At 11 years and 3 months of age, she has had no specific follow-up, and thus undetermined reproductive axis and olfactory clinical status. 

**Figure 2:**
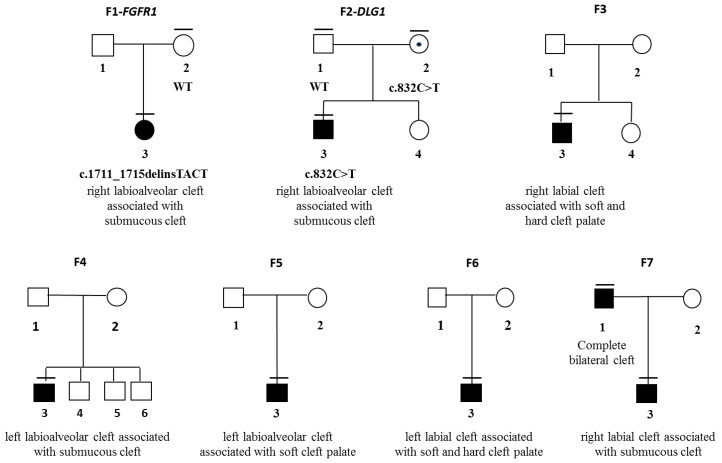
Pedigrees of discontinuous CLP patients. Family number and gene marked above the pedigree (F-#-gene). Gender symbol with a central dot indicates carriers. Variant given underneath each carrier/affected. Clinically studied individuals have a horizontal bar over the top. The CLP phenotype is described in detail.

We identified a 2 bp insertion (c.1711_1715delinsTACT: NM_023110.2) in the *FGFR1* gene. This variant was not found in her mother, and DNA from the father was not available (Figure 2 F1). This variant leads to a frameshift and a premature termination codon (PTC) at position 571 (p.Glu571Tyrfs*61: NP_075598). This insertion is not known in gnomAD v2.1.1 [11]. The gene is intolerant for loss of function (LoF) mutations, evidenced by a probability of being Lof intolerant (pLI) value of one, with four unique predicted truncating alleles out of the 258,000 in the database. The mutation is localized in the αE helix in the FGFR1 tyrosine kinase domain, the essential portion for its catalytic activity. Such a deletion should lead to loss-of-function. Alternatively, this variant may lead to nonsense mediated mRNA decay (NMD), as predicted by Mutation Taster and frequent for PTCs, causing haploinsufficiency.

### 3.2. Patient F2-3

The index case is the first child of two of an unrelated Caucasian couple with no familial history of cleft, born after an uneventful pregnancy. Prenatal diagnosis of right cleft of the lip and alveolus was confirmed at birth. He was born at 39 WG, an Apgar score of 2/7, with the following birth parameters: weight = 3680 g; height = 53 cm; head circumference = 34.5cm. Neonatal cranial, renal and cardiac ultrasounds were normal. Diagnosis of a submucous cleft palate was secondarily made. Apart from frequent upper airway infections, clinical examination at two years of age noted normal growth and development and confirmed the isolated nature of his cleft. Parents’ clinical examination was unremarkable.

A heterozygous nucleotide change (c.832C>T: NM_004087) in exon 10 of the *DLG1* gene was identified in the proband, and segregation analysis showed that the mother was a carrier (Figure 2; F2). This nonsense variant was predicted to cause premature termination of protein synthesis at codon 278 (p.Arg278*: NP_004078) in the first of the three PDZ ( post synaptic disc-large zona) domains. The variant is not present in gnomAD v2.1.1 [11]. The gene is intolerant for LoF mutations as evidenced by a pLI value of 0.99. By deleting most of the functional domains of *DLG1*—PDZ domains, a src-homology 3 (SH3) domain, a band 4.1 domain, and a guanylate kinase homolog (GUK) domain—this variant likely represents loss-of-function. Alternatively, this variant may lead to NMD, as predicted by Mutation Taster (probability: 1), still causing haploinsufficiency.

## 4. Discussion

Phenotypic classification of CLP into three subgroups, defined as CL, CL with CP, and CP, fails to capture discontinuous CLP because “complete” CLP and “discontinuous” CLP cannot be distinguished. As discontinuous CLP is likely included in either the CL or CL/P group, non-syndromic CLP genes have not been specifically implicated in discontinuous CLP. This WES study is the first dedicated to non-syndromic discontinuous CLP. Deleterious mutations were found in two out of seven patients, implicating the *FGFR1* and *DLG1* genes (Table 1). *FGF* signaling plays an essential role in palatal development. Missense and nonsense mutations in the *FGF* gene family, including *FGFR1*, have been noted in a small but significant number of nsCLP cases. Loss-of-function (LoF) mutations in *FGFR1* cause familial and sporadic Kallman syndrome (KS) and normosmic idiopathic hypogonadotropic hypogonadism. The main KS features are anosmia and hypogonadotrophic hypogonadism, and 5% to 10% of these patients have a cleft [26,27]. Missense and nonsense *FGFR1* mutations have also been identified in nsCLP patients [28]. For example, the p.R609X non-sense mutation co-segregated in a family with either KS and CLP or isolated CLP [28]. Patient F1-3 has non-syndromic discontinuous CLP. Following the discovery of this *FGFR1*-likely pathogenic variant, a clinical evaluation focusing on potential hypogonadotrophic hypogonadism and hypo/anosmia has been proposed.

Meta-analysis of GWAS (genome-wide association study) data and a replication study identified the 3q29 chromosomal locus as a cleft-susceptibility locus, with *DLG1* as a candidate gene for nsCL/P [29]. *DLG1* encodes a membrane-associated guanylate kinase protein (MAGUK), a scaffolding protein that is involved in apical–basal cell polarity, cell–cell adhesion and regulation of cellular proliferation. *DLG1* null mutant mice die soon after birth and exhibit major congenital birth defects with craniofacial dysmorphogenesis including cleft palate [30]. *DLG1* is expressed in mesenchymal and epithelial cells throughout the palate, and protein–protein interactions involving the C-terminal domains seem to be essential for the normal function within craniofacial and palatal morphogenesis. It is suggested that loss of DLG1 may affect craniofacial development through Wnt/Planar Cell Polarity Signaling (Wnt/PCP) signalling [31]. Individuals with a 3q29 interstitial microdeletion syndrome, including the *DLG1* gene, have orofacial cleft in 4% to 9% of cases [32]. Moreover, an oral cleft was reported in a patient with a frame shift mutation (c.338_339insTATGTAATTCATAACAATAATAAAT: NM_001098424, p.(E113Dfs*3) in Geno2MP Genotype to Mendelian Phenotype [33]. The variant in patient F2-3 is associated with incomplete penetrance, as his mother is an unaffected carrier.

In the sole reported large cohort of 356 syndromic and non-syndromic discontinuous CLP, four patients were clinically diagnosed as having Van der Woude syndrome (VWS) and one as having popliteal pterygium syndrome (PPS) [8]. Since 70% of VWS syndrome and 97% of PPS have an *IRF6* mutation [34], and taking into account that *IRF6* has been implicated in nsCL/P and nsCP [35,36], *IRF6* is a good candidate gene for discontinuous cleft. Yet, a direct targeted *IRF6* gene sequencing study of four individuals with discontinuous cleft was negative [9]. We did not find any *IRF6* mutations either.

As discontinuous CLP shows considerable variability in terms of affected anatomical structures as reported both in the literature [8] and in our study (Table 1), an even more detailed subgrouping needs to be considered to enable more detailed study of this specific phenotype.

Facial development is a complex multistep process that requires fine spatio-temporal coordination of the expression of numerous genes. Embryonic development of the primary and secondary palate can be subdivided into an early (four to seven weeks of gestation) and late period (seven to twelve weeks of gestation), with different exposures to genes and environmental factors. The anterior palate which extends posteriorly to the incisive foramen or clinically to the incisive papilla, derived from the medial nasal processes, fuses posteriorly with the secondary palate derived from the maxillary processes [7]. Based on epidemiological data and differences in embryological origins, CL/P and CP have historically been considered as distinct entities. Nevertheless, CL/P and CP can segregate in the same family, with etiologic mutations in genes such as *FGFR1, IRF6, MSX1* and *P63* [1], and epidemiologic data suggest that cleft lip only may have unique etiologic features [37]. Moreover, the example of *BMP1a* preferential expression in the primary palate and anterior secondary palate during palatal outgrowth [23] reflects similarities with a spatio-temporal restricted expression pattern, although of different embryological origins. Interestingly, *GREM1*, which encodes a secreted antagonist of *BMP4,* is expressed in the developing lip and soft palate, but not in the hard palate. Variations in the non-coding region near the *GREM1* gene show a highly significant association with discontinuous cleft [24]. Specific mechanisms underlying the occurrence of discontinuous cleft have yet to be explained.

To date, few WES studies have been performed to search for rare coding variants in nsCLP individuals. Moreover, they were mostly applied to familial cases, in which genetic factors are more likely to be prevalent [4,5,38,39,40,41,42,43]. Exome sequencing of 46 nsCLP families unraveled mutations in genes mutated in syndromic forms of CLP in five families (10%), including nsCP families [5]. In the current study, the selection of individuals was exclusively made on the rare discontinuous CLP phenotype, whether familial or not. Following a candidate gene approach, the likely pathogenic variant pick-up rate was high, as truncating variants were found in two genes implicated in nsCLP in two sporadic cases. WES study of homogeneously phenotyped individuals has been one of the keys for identifying genes in rare Mendelian disorders, including syndromic CLP genes [2,44]. Our findings call for accurate phenotyping and stratification in complex disorders, such as nsCLP, and a meticulous clinical evaluation has to be proposed prior to genetic studies (either targeted NGS or WES). WES should be used as the technique of choice as it allows us to identify associated strong mutations for 1/3 of patients. It would also allow further larger analyses in increasing cohorts.

Empiric recurrence risk and the gender ratio are different between non-syndromic CL/P and CP. Substantial evidence is accumulating on rare variants contributing to nsCLP and finding a mutation in a Mendelian CLP disorder can consistently enhance the recurrence risk of nsCLP. Familial studies have been proven to be an effective strategy to identify rare variants and medical screening of such patients has been proposed [5,38,45,46]. Similarly, considering the results in this study, screening patients with discontinuous cleft should be taken into consideration in genetic counseling.

## 5. Conclusions

Whole exome sequencing of clinically well-defined subgroups of CLP, such as discontinuous cleft, is a relevant approach to study CLP etiopathogenesis. It could facilitate more accurate clinical, epidemiological and fundamental research, ultimately resulting in better diagnosis and care of CLP patients. Non-syndromic discontinuous cleft lip and palate seems to have a strong genetic basis.

## Figures and Tables

**Table 1:** Summary of cases.

Patient	Gender	CLP Familial History	Discontinuous CLP Description		Gene, Variant and Protein Change
			Cleft of the Primary Palate	Cleft of the Secondary Palate	
1	F	−	right labioalveolar	submucous	*FGFR1*, c.1809_1810insTC; p.Glu571Tyrfs*61
2	M	−	left labioalveolar	submucous	*DLG1*, c.832C>T; p.Arg278*
3	M	−	right labioalveolar	submucous	
4	M	−	left labioalveolar	submucous	
5	M	−	right labial	soft and hard palate	
6	M	−	left labial	soft and hard palate	
7	M	+	left labial	submucous	

CLP, cleft of the lip and/or palate. Symbols: “F”, female; “M”, male; “−“, negative CLP familial history; “+”, positive CLP familial history.

## Supplementary Materials

The following are available online at https://www.mdpi.com/2073-4425/10/10/833/s1, Table S1: List of 89 studied genes.
