# Supplementary material for: Likely Pathogenic Variants in One Third of Non-Syndromic Discontinuous Cleft Lip and Palate Patients
Source: Genes (Basel). 2019 Oct 22;10(10):833. doi: 10.3390/genes10100833 (PMC6826364; doi:10.3390/genes10100833)
Supplement: Supplementary file 1 [file genes-10-00833-s001.pdf]

Supplementary table 1. List of 89 studied genes

ACTB	GLI2	RBM10
ARHGAP29	GLI3	RUNX1
ATG4C	GRHL3	RYK
BCOR	GRIM2	SATB2
BMP2	HYLS1	SF3B4
BMP4	IRF6	SHH
CDH1	JAG2	SIX3
CHD7	KLF4	SKI
CHRNG	LHX8	SLC26A2
COL2A1	MEIS2	SOX9
COL11A1	MID1	SPECC1L
COL11A2	MLL2	SPRY1
DLG1	MSX1	SPRY2
DLX4	MSX2	SUMO1
DHCR7	NIPBL	TBX1
DHCR24	NECTIN1	TBX22
EFNB1	NUDT6	TBX10
EFTUD2	OFD1	TCOF1
ESCO2	PAX9	TFAP2A
FAF1	PDGFC	TGFB3
FGF8	PHF8	TGFBR1
FGF10	POLR1C	TGFBR2
FGFR1	POLR1D	TGIF1
FGFR2	PQBP1	TP63
FLNA	PROK2	TULP4
FLNB	PROKR2	TWIST1
FOXC1	PTCH1	VAX1
FOXC2	PVR	WNT3
FOXE1	PVRL1	WHSC2
FOXF2	PVRL2	
